# Effects of a Diet Enriched with Polyunsaturated, Saturated, or Trans Fatty Acids on Cytokine Content in the Liver, White Adipose Tissue, and Skeletal Muscle of Adult Mice

**DOI:** 10.1155/2013/594958

**Published:** 2013-08-20

**Authors:** Bruno dos Santos, Debora Estadella, Ana Cláudia Losinskas Hachul, Marcos Hiromu Okuda, Mayara Franzoi Moreno, Lila Missae Oyama, Eliane Beraldi Ribeiro, Claudia Maria da Penha Oller do Nascimento

**Affiliations:** Universidade Federal de São Paulo, Escola Paulista de Medicina (UNIFESP-EPM), 04020-050 São Paulo, SP, Brazil

## Abstract

This study analyzed the effect of diet enriched with 30% lipids on cytokines content in different tissues. Swiss male mice were distributed into four groups treated for 8 weeks with control (C, normolipidic diet); soybean oil (S); lard (L); and hydrogenated vegetable fat (H). We observed an increase in carcass fat in groups S and L, and the total amount of fatty deposits was only higher in group L compared with C group. The serum levels of free fatty acids were lower in the L group, and insulin, adiponectin, lipid profile, and glucose levels were similar among the groups. IL-10 was lower in group L in mesenteric and retroperitoneal adipose tissues. H reduced IL-10 only in retroperitoneal adipose tissue. There was an increase in IL-6 in the gastrocnemius muscle of the L group, and a positive correlation between TNF-*α* and IL-10 was observed in the livers of groups C, L, and H and in the muscles of all groups studied. The results suggested relationships between the quantity and quality of lipids ingested with adiposity, the concentration of free fatty acids, and cytokine production in white adipose tissue, gastrocnemius muscle, and liver.

## 1. Introduction

Epidemiological studies reveal that approximately 2.3 billion adults will be overweight and 700 million will be obese in 2015 [1]. Obesity is a chronic subclinical inflammatory disease of multifactorial etiology, involving the ingestion of a high-calorie diet, a sedentary lifestyle, and genetic predisposition [2]. The establishment of the disease can lead to the development of correlated morbidities such as diabetes mellitus type 2, cardiovascular disease, and metabolic syndrome, among others, as a consequence of the imbalance of a complex system of energy balance [3, 4].

The enlargement of adipocytes and the expansion of adipose tissue lead to local hypoxia and macrophage infiltration, causing an inflammatory response. In turn, the response changes the patterns of protein and gene expression of various bioactive molecules, called adipokines, produced by adipose tissue [5].

Adipokines are a group of proteins synthesized by white adipose tissue that act on the immune, cardiovascular, metabolic, and endocrine systems. Examples include TNF-*α*, leptin, adiponectin, resistin, IL-6, and IL-10 [6, 7]. In obese individuals, the expression of proinflammatory adipokines by adipose tissues is normally increased [8].

TNF-*α* and IL-6 are involved in the development of insulin resistance and atherogenic processes [9]. However, IL-10, a cytokine secreted primarily by monocytes/macrophages and lymphocytes, as well as adiponectin, secreted by adipocytes, possess anti-inflammatory and insulin-sensitizing properties by antagonizing IL-6 and TNF-*α*. Low concentrations of IL-10 and adiponectin have been associated with metabolic syndrome and diabetes mellitus type 2 [10–12]. The IL-10/TNF-*α* ratio has been considered an important indicator of inflammatory status, and low values are associated with an increased risk of morbidity and mortality [13, 14].

The lipid composition of the diet has a strong relationship with the development and persistence of obesity [15]. Acute (2 days) or chronic (16 weeks) treatment with a high-fat diet reduces the synthesis of adiponectin, suggesting that the serum lipid profile and lipid components of the diet are more related to the decrease of adiponectin than to obesity itself [16]. In adult males, a positive correlation between body fat and the quantity or quality of lipids consumed has been described, and diets high in saturated and monounsaturated fatty acids induced greater adiposity compared with diets rich in polyunsaturated fatty acids [17]. 

Studies by our group have shown a link between a high-fat diet and different types of lipids in the diet with changes in gene expression, the synthesis and secretion of adipokines and obesity-related comorbidities [18–21].

Thus, the aim of this study was to investigate the effects of high-fat diets enriched with lard, soybean oil, or hydrogenated vegetable oil (30% lipids, w/w) on body fat content, lipid profiles, serum glucose, insulin and adiponectin concentrations, and the tissue cytokines TNF-*α*, IL-10, and IL-6.

## 2. Materials and Methods

### 2.1. Animals and Diet Protocol

The Experimental Research Committee of the São Paulo Federal University approved all procedures for the care of the animals used in this study (protocol CEP n°1644/10). Thirty-day-old male Swiss mice were used in this study and were kept under controlled conditions (12 h light: 12 h dark cycle at 22°C ± 1°C). During the experimental period, the animals were housed six per cage, receiving water and a specific diet *ad libitum*. The animals were assigned to four experimental groups, with 12 animals per group, according to their specific diet and treated for eight weeks as follows: (1) C (control group; the animals were treated for eight weeks with a standard AIN diet (American Institute of Nutrition); (2) S (soybean oil group); (3) L (lard group); and (4) H (hydrogenated vegetable oil group). The animals' weights were evaluated weekly. 

All diets were prepared according to the recommendations of the AIN [22] and were normoproteic. All groups received a growth diet (AIN-93G) containing 20% protein and at least 7% essential fatty acids between the 30th and 60th day of life and a maintenance diet (AIN-93M) containing 14% protein and 2% soybean oil to provide the essential fatty acids between the 60th and 90th days of life. The compositions of the control, soybean, lard, and hydrogenated vegetable oil diets are described in Table 1.

### 2.2. Fatty Acid Composition of the Diets

500 mg of the prepared diets were used for lipid extraction, saponification, and fatty acid methylation as described by the American Oil Chemists-Society, 2011. The relative fatty acids content in the total lipids from the diet was quantified by gas-liquid chromatography using Agilent Technologies 7890A CG System, with an ionizable flame detector acoplado linked to the EZChrom Elite CDS software (Agilent Technologies, Inc., C.A., U.S.A.). Fatty acids were separated using an SP 2560 capillary column (Supelco, USA), with 100 m × 0.25 mm × 0.20 *μ*m measurements. The chromatographic conditions were similar to those described by Tinoco et al. [23]. The methylated fatty acids were identified by comparing their retention times to known standards (Nu-Chek Prep. Inc). Results were expressed weight percentage (g/100 g of total fatty acids—Table 2).

### 2.3. Experimental Procedure

The animals were sacrificed by decapitation on the 90th day of life in the fasting state (8 h) in the early morning to avoid chronobiological variations. Trunk blood was collected and immediately centrifuged at 4°C, and serum aliquots were taken and frozen at −80°C to measure the concentrations of glucose, triacylglycerols, and total cholesterol using commercials kits from Labtest Diagnostic SA (Minas Gerais, Brazil) and the concentrations of free fatty acids, insulin, and adiponectin using ELISA (Linco Research, Inc., USA). The retroperitoneal (RET), epididymal (EPI), and mesenteric (MES) adipose tissues, gastrocnemius muscle (GAST), and liver were dissected and weighed, frozen in liquid nitrogen, and stored at −80°C until the extraction of protein. The carcasses were weighed and stored for further lipid content determination.

### 2.4. Carcass Lipid Content Determination

The carcasses were eviscerated, weighed, and stored at −20°C. The lipid content was measured as described by Stansbie et al. and standardized using the method described by Oller do Nascimento and Williamson [24]. Briefly, an eviscerated carcass was autoclaved at 120°C for 90 min and then homogenized with double its mass of water. Triplicate aliquots of this homogenate were weighed and digested in 3 mL of 30% KOH and 3 mL of ethanol for at least 2 h at 70°C in capped tubes. After cooling, 2 mL of 12 N H_2_SO_4_ was added, and the sample was washed three times with petroleum ether for lipid extraction. The results are expressed as grams of lipid/100 g of carcass.

### 2.5. Protein Determination and ELISA Assay

Adipose tissue depots, gastrocnemius muscles, and livers (0.25–0.3 g) were homogenized in ice-cold solubilization and total protein extraction buffer (100 mM Tris, pH 7.5, 10 mM ethylene acetic acid, 100 mM sodium fluoride, 10 mM sodium orthovanadate, 2 mM phenylmethylsulfonyl fluoride, 10 mM sodium pyrophosphate, and 0.1 mg/mL aprotinin). After homogenization, Triton X-100 was added to a final concentration of 1%. Samples rested on ice for 30 min and were clarified by centrifugation. Homogenates were centrifuged at 14000 ×g for 40 min at 4°C, the supernatants were saved, and the protein concentrations were determined using a Bradford assay (Bio-Rad, Hercules, California) with bovine serum albumin as a reference. Quantitative assessment of IL-6, IL-10, and TNF-*α* was carried out using ELISA. The results are expressed as pg/*μ*g of protein.

### 2.6. Data Analysis

The results are expressed as the means ± SEM. For multiple comparisons of means, one-way analysis of variance (ANOVA) was performed with subsequent use of the Tukey post hoc test. Simple linear regression analysis was used to evaluate the correlation between cytokines (IL-10 and TNF-*α*). Statistical significance was set at *P* < 0.05.

## 3. Results

### 3.1. Body Weight, Carcass Lipid Content, and Biochemical and Hormonal Serum Analysis

Body mass, white adipose tissue depots (EPI, RET, and MES), liver, and GAST weights were similar among all groups. However, the S and L diets promoted a significant increase in carcass relative lipid content compared with group C. In addition, this parameter in group H was lower than in group S (Table 3). 

Exposure to the L diet after weaning caused an approximately 28% increase in the sum of the adipose tissue depots compared with the control group.

The levels of serum glucose, triacylglycerols, total cholesterol, insulin, and adiponectin did not differ among the studied groups. Nevertheless, the L diet caused a decrease in the serum concentrations of free fatty acids (Table 4). 

### 3.2. Tissue Cytokine Content

**Figure 1:**
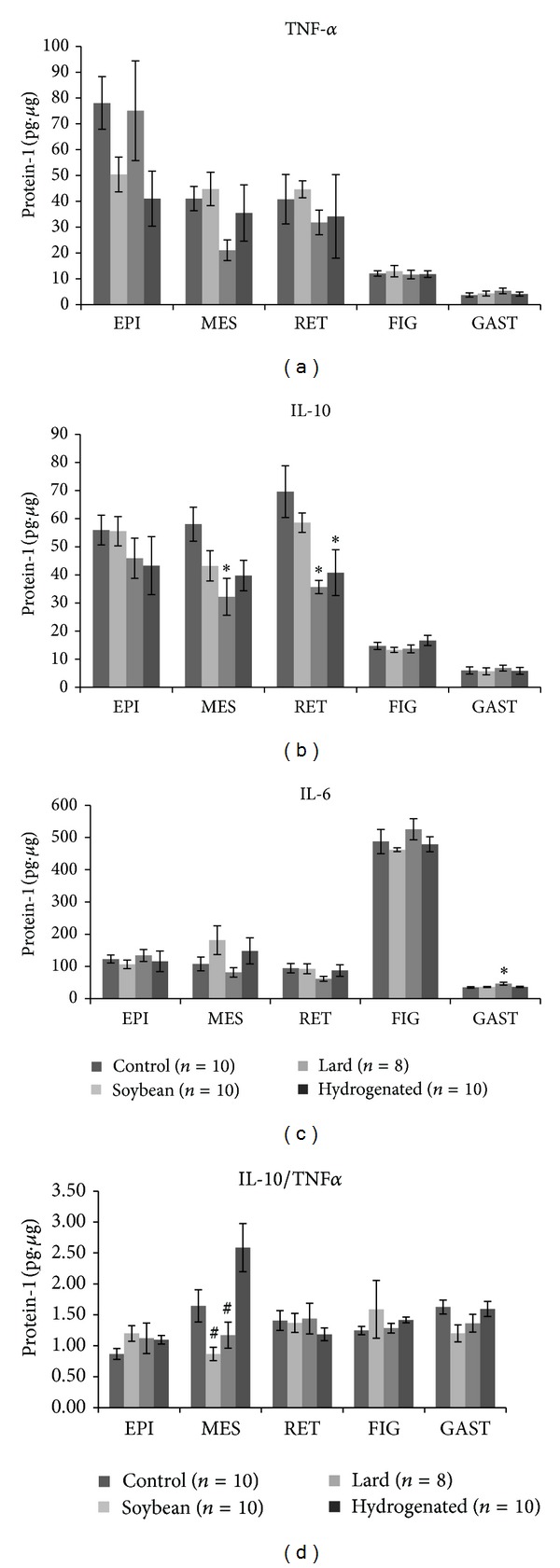
Cytokines levels (IL-6 (pg/ug protein-1) in adipose tissue depots, liver, and gastrocnemius muscle of studied mice groups—(C) control group, (S) soybean, (L) lard, and (H) hydrogenated vegetable fat groups. (a) Tumoral necrosis factor alpha (TNF-*α*), (b) interleukin-10 (IL-10), (c) interleukin-6 (IL-6), and (d) IL-10/TNF-*α* ratio. *Values significantly different from (C) group at *P* < 0.05. ^#^Values significantly different from (H) group at *P* < 0.05.

TNF-*α* levels did not change in any group or tissue studied compared with the control group (Figure 1(a)). 

However, the L diet caused a decrease in IL-10 levels in MES and RET white adipose tissue depots, and the H diet promoted a similar effect in the RET depot (Figure 1(b)). Furthermore, the IL-6 content was elevated in mice that ingested the diet containing saturated fatty acids (L) (Figure 1(c)). In group H, the IL-10/TNF-*α* ratio was higher in MES adipose tissue compared with groups S and L (Figure 1(d)).

We ran correlation tests for IL-10 and TNF-*α* and found the following to be positively correlated: group C, retroperitoneal fat depot, liver, and gastrocnemius muscle; group S, epididymal and mesenteric fat depots and gastrocnemius muscle; and group L, liver and gastrocnemius muscle. In group H, only mesenteric fat tissue did not correlate.

## 4. Discussion

The ingestion of high-fat diets for 8 weeks did not modify body or tissue weight. Similar results were observed by others with eight or seven weeks of high-fat diet treatment [19, 25, 26]. However, other studies have reported an increase in body weight after 16 weeks of high-fat diet treatment [16, 27]. As stated by Noeman et al. [27], the high-fat diet effect is subtle but cumulative, needing at least 10 weeks to increase body weight. These results suggest that the difference in weight gain in the previous studies may be due to the period of treatment.

Despite the fact that the carcass lipid content in groups S and L was higher than in group C, this parameter was similar between groups C and H. The oxygen consumption after a meal was significantly lower in rats fed a lard diet than in rats fed a safflower oil or linseed oil diet [28]. In this sense, Dube et al. [29] demonstrated that the ingestion of a hyperlipidic diet, enriched with lard, caused a decrease in the mRNA expression of UCP-1 in brown adipose tissue. Furthermore, the energy cost of lipid deposition from dietary fat is lower than from dietary carbohydrate [30]. Gaíva et al. [31] demonstrated that enrichment of the diet with polyunsaturated fatty acids caused changes in adipose tissue metabolism, such as the increased uptake of diet-derived lipids favoring fat deposition. Machado et al. [32] reported that mice fed with a trans-fat-enriched diet for 16 weeks showed a reduction in subcutaneous and epididymal fat pads, suggesting an effect on the stimulation of lipolysis. Taken together, these reports suggest that the effects on fat accumulation depend on the type and source of lipids present in the diet and could be related to the effects on energy expenditure and adipose tissue metabolism, such as lipoprotein lipase activity, lipogenesis, and lipolytic enzyme activities. 

The diet intervention did not alter the concentrations of serum glucose, triacylglycerol, total cholesterol, adiponectin, or insulin. Previous studies have demonstrated no effect of high-fat diets on the serum adiponectin concentration [33, 34]. However, a time-dependent effect of a high-fat diet on the adiponectin serum concentration has been shown; 10 weeks of treatment increased and 18 weeks of treatment reduced the serum adiponectin concentration compared with the initial experimental period [35], suggesting a compensatory mechanism to maintain metabolic homeostasis, as this adipokine is associated with the maintenance of carbohydrate and lipid metabolism and also acts to improve insulin sensitivity [19, 36–40]. In fact, in our study, 8 weeks of a high-fat diet treatment did not modify serum glucose, triacylglycerol, total cholesterol, or adiponectin and insulin concentrations, in spite of the increasing adiposity in groups L and S. 

Another possible explanation related to the similar serum lipid profile among the groups is the decrease in the carbohydrate content of the high-fat diets. The review from Volek et al. [41] reported that the serum total cholesterol level remained unchanged from baseline values, while both the HDL and LDL cholesterol levels increased and the TAG levels dramatically decreased under a high-fat diet compared with high-carbohydrate diets. 

In the current study, the lard diet caused a decrease in the serum free fatty acid concentration in relation to the control diet. It has been stated that the plasma free fatty acids resulted from hydrolyzed triacylglycerol in chylomicrons and VLDL by lipoprotein lipase activity and lipolysis in adipose tissue [42]. One important mechanism for the removal of unesterified plasma fatty acids is through oxidation of these fatty acids in skeletal muscle and the heart [43]. Several studies have demonstrated that IL-6 can increase fatty acid oxidation in myocytes [44, 45] and “in vivo” [46]. When we analyzed the IL-6 content in gastrocnemius muscle, we verified an increase in group L compared with group C, which could partially explain the reduction in serum fatty acids in lard-treated animals.

Nutrients such as the fatty acids in special saturated fatty acids can activate intracellular pathways through TLR2 and TLR4, leading to increased proinflammatory gene transcription [47, 48]. In this sense, we analyzed the TNF-*α*, IL-6, and IL-10 concentrations in RET, EPI, MES, gastrocnemius muscle, and liver. The TNF-*α* content was similar among groups in all tissues studied. The literature is controversial about the effects of a high-fat diet on TNF-*α* expression. A similar result was observed by Flanagan et al. [49], who showed that TNF-*α* gene expression in muscle was not significantly affected by the amount or type of dietary fat. 

Hong et al. [50] showed that 10-week-old mice treated with a high-fat diet (55% fat by calories) for 3 weeks had lower IL-10 levels in muscle than control mice, accompanied by a decrease in muscle insulin sensitivity. Furthermore, IL-10 treatment prevents muscle insulin resistance by decreasing obesity-associated macrophages and cytokines in muscle from high-fat diet-treated mice. In the present study, the IL-10 gastrocnemius muscle content did not change in treated mice between 30 and 90 days of life with high-fat diets. However, the lard diet caused a decrease in IL-10 in MES and RET, and the same result was observed in RET from group H without any change in glycemia or insulin plasma concentration, suggesting that the type of diet, age of the animal, and time of treatment could have different effects depending on the tissues evaluated. In addition, we cannot dismiss the possibility that metabolic processes adapt to changes in dietary components during a specific period of life.

The IL-6 level in gastrocnemius muscle increased in group L compared with group C. The role of IL-6 action in obesity-associated insulin resistance remains highly controversial; Wunderlich et al. [51] showed that mice with a hepatic deficiency of IL-6 receptor a (IL-6Ra) develop insulin resistance not only in the liver but also in skeletal muscle and WAT. In addition, the IL-6 level increases with exercise, a situation in which glucose uptake by muscles is high [52]. However, it has been demonstrated that IL-6 administration causes hepatic insulin resistance [53]. 

**Figure 2:**
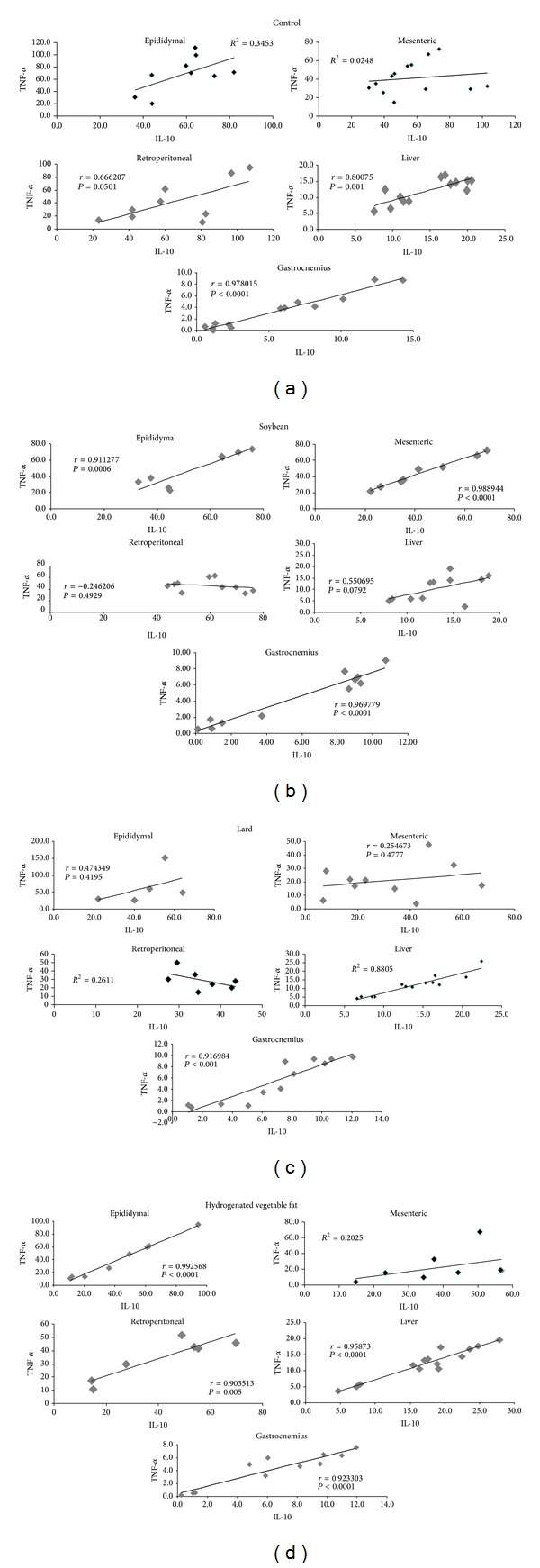
Correlation between cytokines (IL-10 and TNF-*α*) in adipose tissue depots, liver, and gastrocnemius muscle of studied mice groups—(C) control group, (S) soybean, (L) lard, and (H) hydrogenated vegetable fat groups. (Number of samples from 5 to 12 animals).

It is interesting to note that, in the present study, the lard diet, which promoted a decrease in IL-10 in adipose tissue depots, caused an increase in IL-6 in gastrocnemius muscle. From these results, it is possible to suggest that the whole body of an adult animal tries to maintain carbohydrate and lipid metabolism homeostasis by modifying the expression and secretion of cytokines. In fact, by analyzing the ratio between IL-10 and TNF-*α*, which is used as an indicator of the inflammatory condition of the individual (where lower values are associated with a poor prognosis) [13, 14], we verified a similar result among groups in all tissues studied. In addition, a positive correlation between TNF-*α* and IL-10 was observed in the liver (groups C, L and H) and muscle (all studied groups) (Figure 2), which could contribute to the maintenance of glucose, insulin, and lipid profiles at normal values. 

## 5. Conclusion

In summary, our results demonstrate that enrichment of the diet with soybean oil or lard elevated carcass lipid content, whereas enrichment with hydrogenated vegetable oil did not alter this parameter as compared to control diet (4% of soybean oil). Only the lard diet modified the fatty acid serum concentration, decreased IL-10 in adipose tissue depots and increased IL-6 in gastrocnemius muscle. These suggested that the type of fatty acid present in the diet plays a part in determining the amount of carcass lipid content and serum fatty acid concentration and influences the cytokines content in the tissues. 

## Figures and Tables

**Table 1:** Composition of the control (C), soybean (S), lard (L), and hydrogenated vegetable oil (H) diets prepared according to AIN-93.

Ingredient	Diet (g/1000 g)			
	C	S	L	H
Cornstarch	629 (720)	399 (460)	399 (460)	399 (460)
Soybean Oil	70 (40)	20	20	20
Especific lipid	—	280	280	280
Casein	200 (140)	200 (140)	200 (140)	200 (140)
L-Cystine	3 (1.8)	3 (1.8)	3 (1.8)	3 (1.8)
Cellulose	50	50	50	50
Mineral mixture*	35	35	35	35
Vitamin mixture**	10	10	10	10
Bitartrate Choline	2,5	2,5	2,5	2,5
Butylhydroquinone	0.014 (0.008)	0.014 (0.008)	0.014 (0.008)	0.014 (0.008)

The first number refers to the growth diet (AIN-93G), and the number in parentheses refers to the maintenance diet (AIN-93M) when its composition differed from that of the growth diet.

*Mineral mix provided (mg/kg) calcium 5000, phosphorus 1561, potassium 3600, sodium 1019, chloride 1571, sulfur 300, magnesium 507, iron 35, copper 6.0, manganese 10.0, zinc 30.0, chromium 1.0, iodine 0.2, selenium 0.15, fluoride 1.00, boron 0.50, molybdenum 0.15, silicon 5.0, nickel 0.5, lithium 0.1, and vanadium 0.1

**Vitamin mix (mg/kg diet) provided thiamin HCL 6.0, riboflavin 6.0, pyridoxine HCL 7.0, niacin 30.0, calcium pantothenate 16.0, folic acid 2.0, biotin 0.2, vitamin B12 25.0, vitamin A palmitate 4000 IU, vitamin E acetate 75, vitamin D3 1000 IU, and vitamin KI 0.75.

**Table 2:** Fatty acid composition, as percent of total lipid content, of the control (C), soybean (S), lard (L), and hydrogenated vegetable oil (H) diets prepared according to AIN-93.

Fatty acid	Total fatty acids (%)			
	C	S	L	H
C13:0				0,53
C14:0	1,30	0,24	1,14	0,21
C16:0	11,28	10,37	22,70	12,36
C16:1 9t			0,29	
C16:1 9c		0,13	1,66	0,09
C17:0		0,14	0,37	0,14
C17:1 10c			0,25	0,06
C18:0	3,48	3,48	14,33	14,43
C18:1 6t+8t				4,77
C18:1 9t			0,09	4,66
C18:1 10t				6,50
C18:1 11t			0,10	5,29
C18:1 13t+14t				3,25
C18:1 9c	16,59	23,32	36,38	22,55
C18:1 11c	2,06	1,51	2,36	1,11
C18:1 12c				2,59
C18:1 13c				4,53
C18:1 14c				0,59
C18:1 15c				0,20
C18:1 16t				0,21
C18:2 9c,12c (w6)	45,60	45,90	16,87	11,36
C18:2 9t,12t		0,24		
C18:2 9t, 12c				0,40
C18:2 11t, 15c				0,14
C18:2 9t, 13c+8t, 12c				0,40
C18:3 6c,9c,12c (w6)		0,30	0,05	0,08
C18:3 9c,12c,15c (w3)	6,87	5,11	1,67	1,21
C19:0			0,09	0,27
C19:1 7c			0,08	0,22
C20:0		0,41	0,28	0,57
C20:1 8c		0,15		0,06
C20:2 11c,14c		0,05	0,61	0,09
C20:4 5c,8c,11c,14c (w6)		0,08	0,26	0,09
C22:0		0,55		0,68
C22:4 7c,10c,13c,16c	12,83	7,61		0,21
C24:0		0,24		0,23

	Fatty acids (g/100 g diet)			

SFA	0,64	4,63	11,67	8,83
MUFA-cis	0,75	7,50	12,22	9,6
MUFA-trans	0	0	0,14	7,40
PUFA-cis	2,61	17,70	5,8	3,91
PUFA-trans	0	0,07	0	0,28

MUFA: monounsaturated fatty acids; ND: not detected; PUFA: polyunsaturated fatty acids; SFA: saturated fatty acids.

**Table 3:** Body Mass (g), carcass relative lipid content (g/100 g), sum of adipose tissue depots (g/100 g) and tissue weight (g/100 g) of studied mice groups-(C) control group, (S) Soybean, (L) Lard and (H) Hydrogenated vegetable fat groups.

Variables	Groups			
	C	S	L	H
Weight				
Initial	22.09 ± 1.08	22.36 ± 0.93	22.87 ± 0.52	22.08 ± 0.51
Final	48.17 ± 1.83	51.31 ± 1.92	52.55 ± 2.18	51.70 ± 2.18
Carcass relative lipid content	21.82 ± 0.85	31.59 ± 2.18*	30.28 ± 3.57*	22.02 ± 1.95^+^
EPI	3.70 ± 0.25	4.23 ± 0.20	4.69 ± 0.29	4.43 ± 0.29
RET	0.94 ± 0.05	1.22 ± 0.08	1.21 ± 0.12	0.94 ± 0.06
MES	2.01 ± 0.19	2.41 ± 0.25	2.45 ± 0.21	2.28 ± 0.24
Sum of adipose tissue depots	6.53 ± 0.42	7.87 ± 0.35	8.35 ± 0.49*	7.66 ± 0.45
Liver	3.62 ± 0.12	3.76 ± 0.33	3.66 ± 0.15	3.69 ± 0.14
GAST	0.60 ± 0.06	0.56 ± 0.06	0.56 ± 0.05	0.54 ± 0.04

All results are presented as means ± standard error of the mean (*n* = 9).

*Values significantly different from (C) group at *P* < 0.05.

^+^Values significantly different from (S) group at *P* < 0.05.

**Table 4:** Serum triacylglycerols (mg/dL), total cholesterol (mg/dL), free fat acids (*μ*M), insulin (ng/mL), adiponectin (*μ*g/mL) and glucose (mg/dL) of studied mice groups-(C) control group, (S) Soybean, (L) Lard and (H) Hydrogenated vegetable fat groups.

Variables	Groups			
	C	S	L	H
Triacylglycerols	182 ± 7.88	165 ± 7.67	158 ± 5.54	168 ± 5.12
Total cholesterol	158 ± 7.09	147 ± 5.38	143 ± 4.79	160 ± 15.66
Free fat acids	2.80 ± 0.16	2.26 ± 0.13	1.77 ± 0.24*	2.15 ± 0.25
Insulin	1.60 ± 0.33	1.45 ± 0.13	0.93 ± 0.18	2.02 ± 0.38
Adiponectin	3.46 ± 0.28	4.14 ± 0.25	3.17 ± 0.39	3.45 ± 0.39
Glucose	120 ± 7.68	130 ± 11.14	125 ± 13.44	112.0 ± 10.52

All results are presented as means ± standard error of the mean (*n* = 8).

*Values significantly different from (C) group at *P* < 0.05.
